# Thoracoscopic Ablation of Critically Located Liver Tumors: A Safety and Efficacy Cohort Study

**DOI:** 10.3389/fsurg.2021.626297

**Published:** 2021-03-17

**Authors:** Umberto Cillo, Michele Finotti, Chiara Di Renzo, Alessandro Vitale, Giacomo Zanus, Enrico Gringeri, Alessandra Bertacco, Marina Polacco, Francesco D'Amico

**Affiliations:** ^1^Department of Surgery, Oncology and Gastroenterology, Hepatobiliary Surgery and Liver Transplantation, Padova University, Padova, Italy; ^2^Department of Surgery, Immunology and Transplantation Unit, Yale University, New Haven, CT, United States

**Keywords:** hepatocellular carcinoma, colon rectal liver metastases, microwave ablation, minimal invasive treatments, thoracoscopic liver ablation, trans-diaphragmatic approach

## Abstract

**Background:** Liver resection represents the first curative treatment to treat primary and secondary hepatic tumors. Thoracoscopic liver ablation is a viable and minimally invasive alternative treatment, especially for patients with previous multiple abdominal surgeries. The aim of the study was to evaluate the safety and efficacy of thoracoscopic ablation for liver tumors.

**Methods:** Retrospective analysis of a prospective database of patients with liver tumors, treated with thoracoscopic trans-diagrammatic ablation (MWA or RFA) at our institution from 2012 to 2018. The primary endpoint was post-operative mortality at 30 days, while secondary endpoints were morbidity and efficacy of ablation (i.e., response rate evaluated according to mRECIST criteria, and overall patient survival). Patient demographics, operational characteristics, and complications were recorded.

**Results:** A total of 13 nodules were treated in 10 patients with a median age of 65.5 years. Post-operative mortality was 0%, and overall morbidity was 40% (Clavien-Dindo I complications 30%, II 0%, III 10%, IV 0%). Complete radiological response was obtained in 83.3% of nodules at 3 months. After a median follow-up of 20.95 months, the local tumor progression rate was 30%, with an intra-segmental-recurrence of 30%, and an intra-hepatic-recurrence of 30%. The overall 1-, 2-, and 3-years survival rates were 80%, 58%, and 58%.

**Conclusion:** Thoracoscopic trans-diaphragmatic ablation proved to be a safe and effective way to treat liver tumors when abdominal approach is not feasible. Considering the low morbidity, it is a viable option to treat patients with recurrent disease and/or previous multiple abdominal surgeries.

## Introduction

Liver resection represents the first curative indication to treat primary and secondary hepatic tumors (1). However, surgical excision is not always feasible. Liver resection in patients with numerous comorbidities, poor liver function due to cirrhosis, and/or multiple previous surgeries, is associated with higher mortality and morbidity (2).

As alternative therapies, minimally invasive treatments, such as laparoscopic or percutaneous RadioFrequency (RFA) and MicroWave (MWA) ablation, have been gaining interest (3).

Loco regional treatments are considered safe and effective in the treatment of HCC and liver metastases (4). However, for hepatic tumors located beneath the diaphragm and/or in the hepatic dome (segments 7, 8, and 4A), or in patients with multiple previous abdominal surgeries, conventional laparoscopic or percutaneous approaches are demanding, due to the difficulties related to tumor identification.

In this scenario, a trans-thoracic and/or a thoracoscopic ablation has been proposed.

Most clinical data of trans-thoracic/thoracoscopic liver tumor ablation are based on experience with RFA and have short follow-ups (5). The use of MWA to treat liver tumors is less investigated despite several theoretical advantages compared to RFA. MWA ablation, with a shorter operative time, allows a larger ablation area and higher temperature. Moreover, MWA ablation is not affected by the so-called heat-sink effect and multiple probes can be used at the same time (6–9).

We describe, to the best of our knowledge, the largest series of hepatic tumors treated with a thoracoscopic approach with RFA or MWA ablation, in terms of specific safety and efficacy endpoints. Moreover, we report a detailed literature review on this specific field.

## Materials and Methods

### Patients

A retrospective analysis conducted on a longitudinal, prospectively collected database of identified patients with liver tumors treated with thoracoscopic trans-diaphragmatic ablation (RFA or MWA) from January 2012 to March 2018 at the Hepato-Biliary Surgery and Liver Transplantation at Padua University Hospital. No IRB approval was needed for this study.

The inclusion criteria were as follows:

1. Ineligibility for liver resection due to: critical position near major hepatic structures (hepatic veins), insufficient future liver remnant after resection, and/or poor liver function (Child score B/C)
2. Nodules critically approachable with abdominal ablation (open, laparoscopic, or percutaneous) due to previous abdominal surgery (multiple laparotomies) or due to location in the hepatic dome (Sg.s VII; VII; IVA)
3. Permissive respiratory function
4. No extra hepatic disease
5. Hepatocellular carcinoma (HCC) <5 cm, intrahepatic cholangiocarcinoma (iCCA) and ColoRectal Liver Metastases (CRLMs) <3 cm.

Previous thoracic surgery was not an absolute contraindication to the procedure.

To reduce confounding factors, nodules previously treated with other loco regional therapies were excluded from the analysis.

### Pre- and Post-Surgical Evaluation

Once the diagnosis of liver tumors was suspected, a complete staging of the disease was done as follows:

1. CT/MRI scan of chest/abdomen and pelvis.
2. PET-CT was performed in clinical suspect of extra hepatic disease.
3. Laboratory tests: blood chemistry, hepatic, renal, coagulation function, and tumor biomarkers (CA125, CA19-9, CA 15-3, CEA).

After hospital discharge, all patients were followed up at the outpatient clinic with physical examinations, biochemical liver function tests, tumor markers, CT, and/or MRI at 1 month and then repeated every 3 months for the first 2 years.

### Surgical Procedure

The main goal of minimally invasive thermal ablation (RFA or MWA) is to destroy the malignant cells using heat without damaging adjacent vital structures. MWA were performed using a 2.45-MHz generator (AMICA-GEN, © 2020 H.S. Hospital Service S.p.A., Aprilia, Italy) delivering energy through a 14- or 16-gauge internally cooled coaxial antenna (AMICA PROBE, © 2020 H.S. Hospital Service S.p.A, Aprilia, Italy), featuring a miniaturized quarter wave impedance transformer (referred to as a mini choke) for reflected wave confinement, as previously reported (3). The radiofrequency (RF) ablation techniques were performed using a Cool-tip™RFA (Medtronic, Minneapolis, MN, USA).

**Figure 1:**
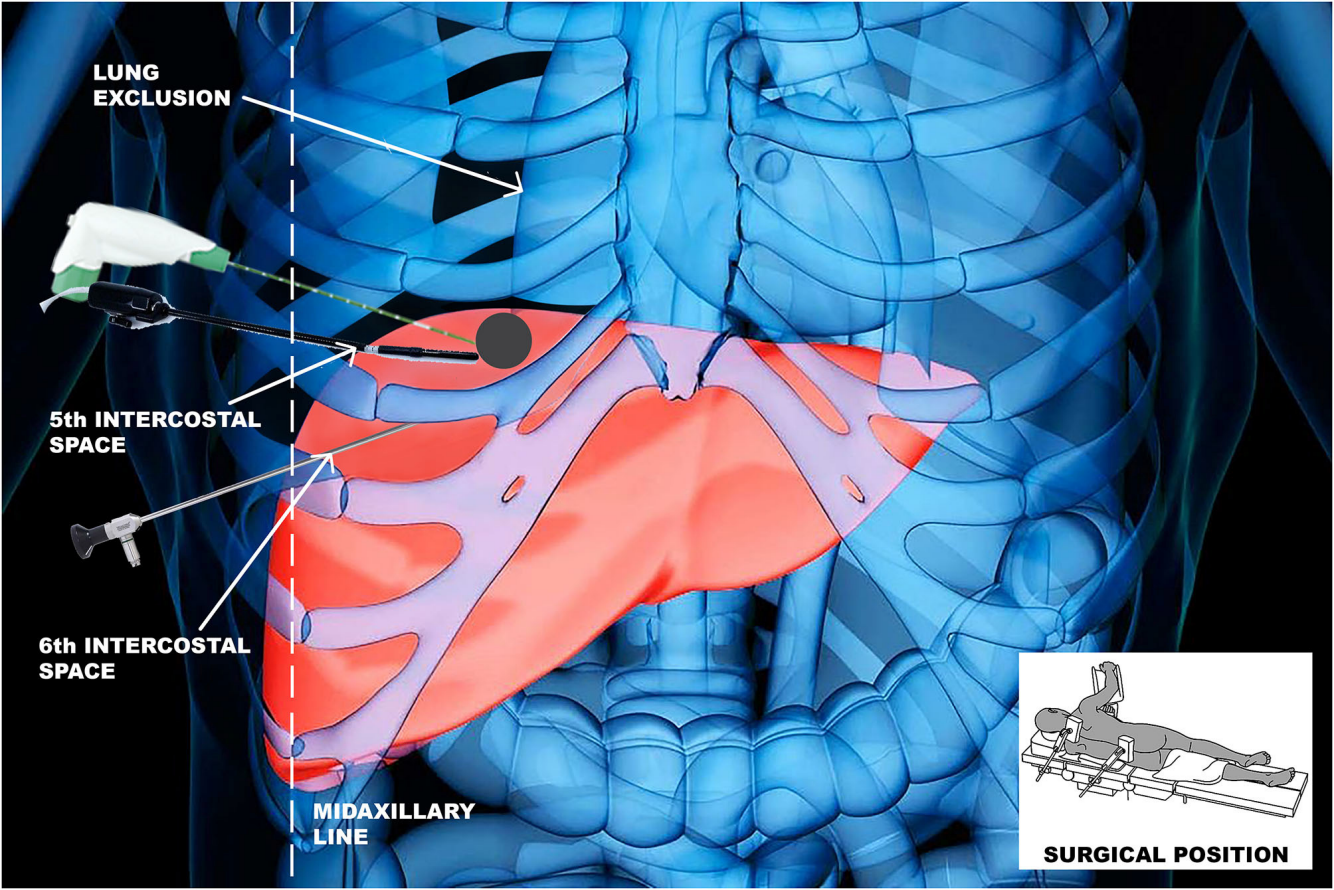
The surgical procedure with a thoracoscopic trans-diaphragmatic approach to perform tumor ablation at the liver dome.

The surgical procedures were performed with a thoracoscopic trans-diaphragmatic approach; the patient was placed in a left lateral and mild anti-Trendelenburg position, in consideration of the location of nodules in the right hepatic dome. Double-lumen tube intubation was necessary in order to exclude right lung ventilation during the procedures. Usually, two or three trocars were placed in the fifth and sixth intercostal space, for optic and US evaluation. Under vision of the diaphragm, triangulating the liver nodules across the diaphragm with US, we positioned the MW/RFA needle with an approach trans-thoracic through the diaphragm (Figure 1).

### Study Endpoints

#### Primary Endpoint

1. Safety of thoracoscopic trans-diaphragmatic ablation: post-operative mortality at 30 days.

#### Secondary Endpoints

1. Morbidity profile (according to the Clavien-Dindo classification) (7); days of hospitalization; total operative time; ICU long of stay.
2. Radiological response rate: Local tumor progression (LTP), Intra-segmental recurrence (ISR), and intrahepatic recurrence (IHR) rates (m-RECIST Criteria)
3. Overall Survival (OS)
4. Disease-free rate and Overall recurrence rate.

### Response Rate Evaluation

Local tumor progression (LTP) was defined as the persistence of active, enhancing tissue within or adjacent (2 cm) to the ablation site (4).

Intra-segmental recurrence was defined as the occurrence of tumor nodules in the same liver segment where ablation had been performed (4), but distant from the ablation site.

Intrahepatic recurrence (IHR) was defined as the appearance of tumor nodules in other liver segments (apart from any LTP or ISR) (4).

To evaluate the effectiveness of the ablative technique, a CT or MRI abdomen with contrast were performed at 3 months using modified-RECIST (m-RECIST) criteria. Two different and independent radiologists evaluated the imaging. M-RECIST criteria were used to evaluate treatment efficacy (8).

### Statistical Analysis

Values for continuous variables are presented as medians (ranges). Values for categorical-nominal variables are presented as frequencies (%).

The length of the follow-up after thoracoscopic trans-diaphragmatic ablation was calculated from the date of the operation to the date of HCC recurrence (for disease-free survival analysis), the patient's death (for survival analysis), or the latest follow-up. The last follow-up date considered was 31 December 2019. The length of follow-up and survival were expressed as median (range). Overall survival curves were calculated using the Kaplan–Meier technique and compared with the log-rank test. A *P* < 0.05 was considered to indicate statistical significance. All statistical calculations were performed using jmp Version 9.0 2010 (SAS Institute, Inc., Cary, NC, USA).

### Characteristics of Patients and Procedures

From January 2012 to March 2018, a total of 10 consecutive thoracoscopic ablation procedures on 10 patients were performed in our institution.

A total of 13 liver tumors were ablated. Eight HCC nodules in seven cirrhotic patients, four iCCA nodules in two patients, and one CRLMs nodule in one patient were treated (Table 1). Two nodules were ablated with RFA and 11 with MWA ablation technique.

**Table 1:** Characteristics of patients and previous treatments.

**Variables**		
Age	Median 65.5 years (range 59–74)	
Sex	Males: 9	
	Females: 1	
Type of primary tumor	HCC: 8 iCCA: 4 CRLM: 1	
Number of nodules	Median 1 (range 1–3)	1 nodule: 8 patients 2 nodules: 1 patient 3 nodules: 1 patient
Tumor size	Median 21 mm (range 4–46) HCC: median 20.5 mm (range 10–46) iCCA: median 14.7 mm (range 4–25) CRLM: 25 mm	
Approach	Thoracoscopic ablation	
Location of the nodules	S8: 6 nodules	
	S7: 6 nodules	
	S4: 1 nodules	
Previous surgery	100% (10 patients)	
Type of previous surgery	Thermal ablation	70%
	Liver resection	80%
	Liver Transplant	20%
	Lung resection	10%
Other procedures	TACE (30%), hemicolectomy (10%)	

*HCC, hepatocellular carcinoma; iCCA, intra-hepatic cholangiocarcinoma; CRLM, colorectal liver metastasis; S, segment; TACE, trans-arterial chemoembolization*.

Median age was 65.5 years (range 59–74 years) with nine males and one female.

All patients received at least one abdominal surgery before our procedure (Table 1). Most patients had been through multiple laparotomies, especially for liver resection, thermal ablation, or liver transplantation. To note, one patient had previous thoracoscopic surgery for a biopsy of a nodule in the right apex, with a condition of minimal adhesion in the right chest, not precluding the procedure.

## Results

Median tumor size was 21 mm (range 4–46 mm). A single nodule was treated in 80% of the procedures, while multiple nodules (range 1–3) were treated in 20% of the procedures. Most nodules were in the liver segments seven and eight (90.9%). The median total operating time was 120 min (range 40–225 min) and the mean ablation time per nodule was 8 min (range 3–24 min) at a median power of 40 Watt (range 40–60 Watt) for MWA and 100 Watt for RFA. The characteristics of procedures and their complications are described in Table 2.

**Table 2:** Characteristics of procedures and complications.

**Variables**	**MWA 8 patients**	**RFA2 patients**
Time of ablation (minutes)	Median 8 min (range 5–15 min)	Median 24 min^*^
Power (Watt)	Median 40 Watt (range 40–60 Watt)	Median 100 Watt^*^
Length of operation (minutes)	Median 85 min (range 40–225 min)	Median 143 min (range 120–165 min)
Post-operative length (days)	Median 6 days (range 3–20)	Median 9 days (range 6–11)
Complications Clavien-Dindo classification	MWA 8 patients	RFA 2 patients
C-D I: 3 patients (30%)	C-D I: 2 Pleural effusion, subcutaneous emphysema and PNX	C-D I: 1 Pleural effusion
C-D II: 0	C-D II: 0	C-D II: 0
C-D III: 1 patients (10%)	C-D III: 1 Biliary fistula requiring explorative laparotomy	C-D III: 0
C-D IV: 0	C-D IV: 0	C-D IV: 0
C-D V: 0	C-D V: 0	C-D V: 0

* *The 2 RFA have 24 min time of ablation and 100 W power. MWA, microwave ablation; RFA, radiofrequency ablation; C-D, Clavien Dindo; PNX, pneumothorax*.

### Safety

Regarding the primary endpoint of this study, we did not observe any intra or post-operative death (Table 2). All patients positioned a chest drainage that was removed on post-operative day 3. The median hospital stay was 7 days (range 3–20 days), and no patient required ICU stay. The overall morbidity rate according to Clavien-Dindo classification (Table 2) was 40%, with grade I complications occurring in 30% of patients, and grade III in 10% (one patient). The most common complication was a mild pleural effusion (Clavien Dindo I) occurring in three patients and probably related to a thermal-ablation inflammation of the diaphragm, associated with other low-risk complications: pneumothorax <2 cm in one patient and subcutaneous emphysema in one patient. Only one patient required a laparotomic surgical revision due to a post-operative biliary fistula that prolonged post-operative stay (20 days) that was treated by closing the needle track in the liver with a laparotomic approach. We did not observe any grade IV complications.

### Efficacy

According to mRECIST classification, a complete radiological response was achieved in 83.3 and 62.5% of the nodules, respectively, at 3 months and 1 year.

**Figure 2:**
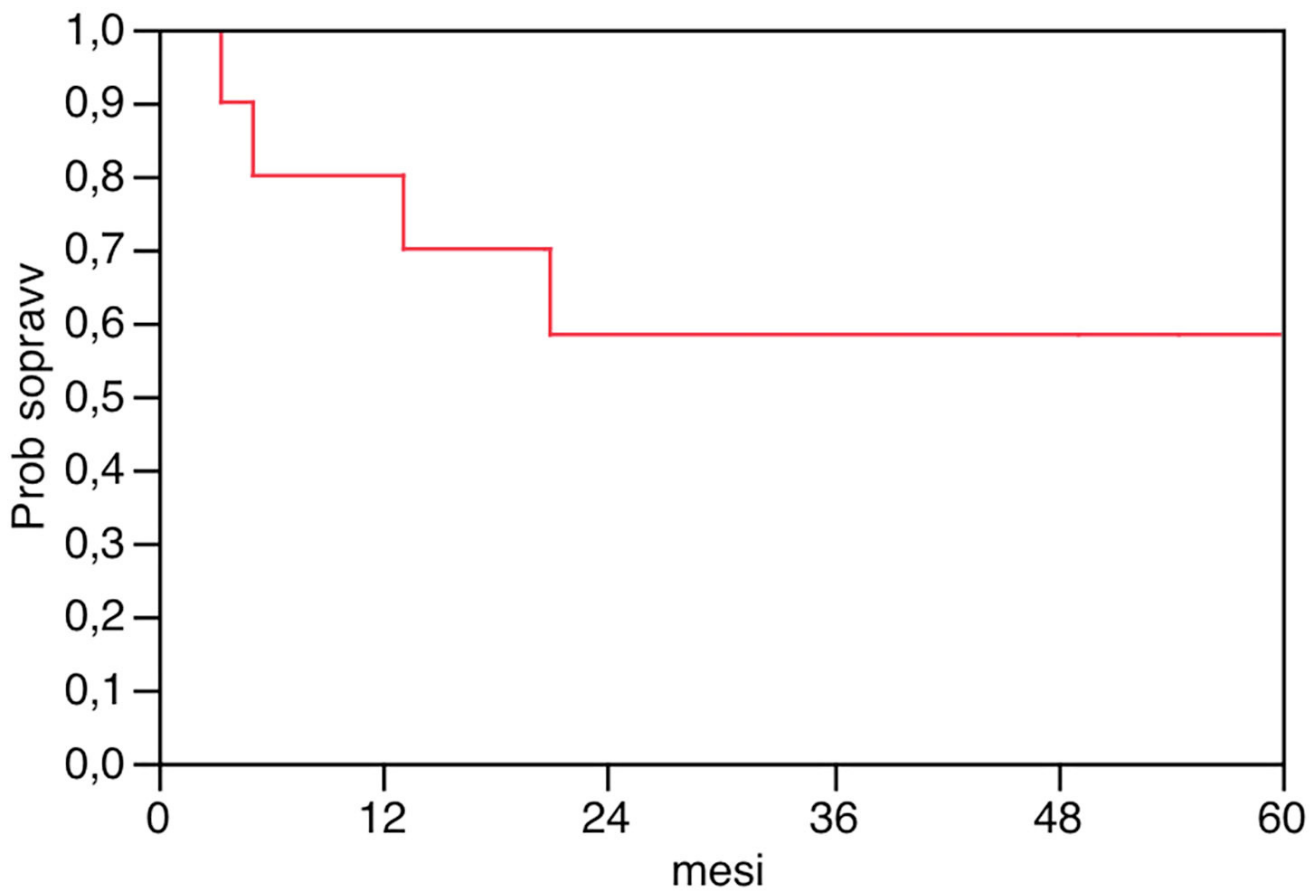
Overall survival after thoracoscopic thermal ablation in 10 patients.

After a median follow-up of 20.95 months (range 3.37–94.97), the overall disease-free survival rate was 60%, the LTP was 30%, the ISR was 30%, the IHR of 30%, and the EHR was 30% (Table 3). The overall survival at 1, 2, and 3 years was 80, 58, and 58%, respectively (Figure 2). Median survival was not reached.

**Table 3:** Response rate evaluation.

**Variable**	**13 nodules**
Overall recurrence rate	40%
• LTP rate	30%
• ISR rate	30%
• IHR rate	30%
• EHR rate	30%
Disease free rate	60%

*LTP, local tumor progression; ISR, intra-segmental recurrence; IHR, intra-hepatic recurrence; EHR, extra-hepatic recurrence*.

## Discussion

Surgical resection is the gold standard to treat resectable liver tumors. The benefit in terms of overall survival after complete resection is well-recognized and established. However, liver resection is not always feasible, often due to patient comorbidities, disease stage, and/or poor liver function (10).

Loco regional treatments, such RFA and MWA, have been considered possible alternative therapies (11). Recently, in unresectable liver tumors (especially HCC and CRLM), national and international guidelines considered local therapies as a practicable alternative to resection (4).

For liver tumors located in the hepatic dome, a lateral thoracic approach has been proposed (thoracotomy, thoracoscopy, or trans-thoracic percutaneous). Thoracotomy is usually indicated in case of liver resection, but it is an invasive procedure not always feasible in fragile patients. Trans-thoracic percutaneous, US or CT guided, has been shown to be effective and safe (12, 13). However, tumors located in difficult positions can be challenging to detect with a percutaneous approach, and risk related to the induce pneumothorax (especially in patient with previous thoracic surgery) and air embolism secondary to lung penetration have been described (14). A combined approach has been proposed, using an abdominal laparoscopic approach plus trans-thoracic percutaneous. However, in case of patients with previous multiple abdominal surgeries, the combined approach is not always safe and possible (15).

In the present study we considered the thoracoscopic approach as the best-balanced alternative between the thoracotomy and trans-thoracic percutaneous approach, especially for patients fit for a loco regional treatment, but not for open surgery, and with multiple previous abdominal surgeries. We preferred the use of MWA, associated with some theoretical advantages compared to RFA (6), despite the lack of definitive evidence supporting one or the other.

Our series underlines the indication of minimally invasive treatment in patients with high risk of morbidity and mortality: the median age of the patients was 65.5 years old. All patients had previous abdominal surgeries and liver treatments, especially liver resections or open liver ablations. One patient was treated for a recurrent HCC after liver transplantation, since the absence of adjuvant therapy to prevent tumor recurrence in this specific setting is well-known (16).

It is important to emphasize that this cohort of patients would not be fit for other procedures, due to their comorbidity and previous surgical therapies. The thoracoscopic approach allowed a potentially radical therapeutic chance with no mortality and low morbidity.

Indeed, despite this high-risk patient selection, the overall intra and peri-operative mortality was 0 and the overall morbidity was 40%; most complications (30%) were minor (grade I), and only one patient had grade III complications.

In several observational studies, complications after MWA ranged between 0 and 54% (17–21).

Factors associated with the safety of the minimally invasive approach were the short operative time (median of 120 min), the possibility to treat more than one nodule in the same procedure, and the relatively rare occurrence of severe complications after surgery (only one patient had a Dindo Clavien 3).

Comparable with the literature, in our series the most common complications were pleural effusion, pneumothorax, and subcutaneous emphysema. Diaphragmatic hernia due to heat injury, a possible fatal complication, did not occur.

The first series of thoracoscopic liver ablation was described in 1998 by Yamashita et al. (22) (Table 4). Six HCC nodules were treated with MWA technique, with low morbidity and mortality. This study, even if LTP, IHR, and EHR had not been described in detail, showed that the thoracoscopic approach is a viable option to treat liver tumors in the hepatic dome, especially in patients with poor liver function.

**Table 4:** Morbidity, mortality, and recurrence after thoracoscopic liver ablation: review of the literature and our experience.

**References and year**		***N*** **of patients**		**Type**	**Mean *n*. nodules**	**Mean size (mm)**	**Mean LOS (days)**	**PO mortality (%)**	**Morbidity ≥ III (%)**	**FU (months)**	**LTP (%)**	**IHR (%)**	**3 y OS (%)**
Yamashita et al. (22)	‘98	6	4 HCC	MWA	1	11–22	10.5	0	0	4–23	0	0	–
			2 CRLM										
Ishikawa et al. (23)	‘01	9	HCC	• 8 MWA 1 RFA	1	17.2	–	0	11	25	22–56	–	–
Lee et al. (24)	‘04	3	1 CCA	RFA	2	40	7	0	0	8	0–67	–	–
			1 CRLM										
			1 metastatic melanoma										
Kurokohchi et al. (25)	‘06	6	HCC	RFA	–	10–25	–	–	–	6.6	0	–	–
Padua experience Present series	20	10	7 HCC	2 RFA 8 MWA	1 (1–3)	21 (4–46)	7	0	10%	20.95 (3.37–94.97)	30%	30%	58%
			2 iCCA										
			1 CRLMs										

In 2001, Ishikawa et al. treated nine HCC nodules with good outcome: only one patient had major pleural bleeding (23).

Lee at al. In 2004 reported zero mortality and morbidity in three patients treated with a RFA thoracoscopic approach. In the follow-up, one patient was lost and the other two patients died of progressive metastatic disease at 8 and 20 months, respectively (24).

Thoracoscopic ablation with RFA associated with ethanol injection has been proposed: Kurokohchi et al. treated six HCC patients with no local recurrence, but a short follow-up was described (6.6 months) (25).

Recently, only case reports with thoracoscopic RFA have been reported (26, 27).

The thoracoscopic approach has also been evaluated in association with other procedures.

A combined laparoscopic abdominal liver resection and thoracoscopic ablation have been proposed, according to the tumor liver location. The laparoscopic approach allowed for better parenchymal and vascular control during the liver thoracoscopic resection and/or ablation, with low post-operative complications (15, 28, 29).

This type of technique can be used to minimize the invasiveness of open liver resection, but the laparoscopic abdominal approach is not always feasible in patients with poor liver function and multiple previous surgeries.

Fujiwara et al. evaluated the safety and efficacy of CT-guided RFA ablation for sub diaphragmatic HCC (14). The procedure was proposed with an induced artificial pneumothorax to reduce the risk of lung penetration related to percutaneous approach. This technique achieved good results in term of ablation with low complications. However, the limitations of the percutaneous approach persisted and patients with previous thoracic surgeries would not be suitable for this procedure.

### Limitations

Our study has a number of limitations. First, even though it is based on a prospectically collected dataset, it is a retrospective study. Second, the low number of procedures limits the possibility to draw definitive conclusions. The process of patient superselection justifies such a low numerosity in the present study and in the previous ones. In this sense, the thoracoscopic tool may represent a “niche” therapy to increase the armamentarium aimed at potential radicality. On this perspective, however, we have to stress that enrolled patients had only palliative solutions as therapeutic alternatives. Third, series inhomogeneity including HCC, CCA, and CRLM may act as a confounding factor, in particular in the interpretation of data related to prevalence of complete ablation and disease-free survival. Finally, two different ablation techniques have been used in treating liver nodules: MWA and RFA.

*All the above-mentioned limitations prompt the need to carry out further prospective multicenter studies including an adequate number of homogeneously stratified patients*.

To the best to our knowledge, we described the largest series of thoracoscopic liver tumors' ablation with MWA or RFA. For the first time, local tumor progression and OS are described in detail with the longest follow-up in literature (Table 4).

The present series supports the use of minimally invasive thoracoscopic ablation as a viable alternative treatment for liver tumor, to implement the therapeutic armamentarium of critically located liver tumors. The procedure was associated with a good local tumor control and with low risk of peri- and post-procedural complication. In patients with high comorbidities and previous multiple abdominal surgeries, the treatment was safe and enabled the opportunity to achieve oncologic radicality. Patient selection is a crucial step for thoracoscopic ablation: our series reveals that tumor dimension and liver function have been associated with overall survival, disease-free rate, and complete nodules response at 1 year.

In the thoracoscopic approach, compared to percutaneous treatment, hepatic dome lesion can be better recognized; an extended intra operative liver ultrasound can be easily performed and an optimal visualization and control of the thermal ablation procedure can be achieved, especially for lesions in critical locations and/or lesions near the heart or diaphragm.

## Data Availability Statement

The raw data supporting the conclusions of this article will be made available by the authors, without undue reservation.

## Ethics Statement

Ethical review and approval was not required for the study on human participants in accordance with the local legislation and institutional requirements. The patients/participants provided their written informed consent to participate in this study.

## Author Contributions

UC and FD'A: conceptualization, methodology, writing—review, and editing. MF: data curation and writing—original draft. CDR: data curation. AV: data curation, validation, review, and editing. GZ, EG, AB, and MP: validation. All authors have read and agreed to the published version of the manuscript.

## Conflict of Interest

The authors declare that the research was conducted in the absence of any commercial or financial relationships that could be construed as a potential conflict of interest.

## Abbreviations

CRLM: Colon Rectal Liver Metastases
CR: Complete Response
DFS: disease free survival
EHR: ExtraHepatic Recurrence
HCC: HepatoCellular Carcinoma
IHR: IntraHepatic Recurrence
ISR: Intra Segmental Recurrence
LTP: Local Tumor Progression
MWA: microwave thermal ablation
OS: Overall Survival
RFA: Radiofrequency Ablation.
